# A prospective interventional study investigating sleep disorders prior to and during adjuvant radiotherapy for breast cancer

**DOI:** 10.1186/s12885-021-09084-w

**Published:** 2021-12-20

**Authors:** Dirk Rades, Carlos A. Narvaez, Liesa Dziggel, Stefan Janssen, Denise Olbrich, Soeren Tvilsted, Troels W. Kjaer

**Affiliations:** 1 Department of Radiation Oncology, University of Lübeck, Ratzeburger Allee 160, 23562 Lübeck, Germany; 2 Department of Radiation Oncology, University of Lübeck, Lübeck, Germany; 3 Department of Radiation Oncology, University of Lübeck, Lübeck, Germany; 4 Department of Radiation Oncology, University of Lübeck, Lübeck, Germany; 5 The Centre for Clinical Trials Lübeck, Lübeck, Germany; 6 Research Projects and Clinical Optimization, Zealand University Hospital, Koege, Denmark; 7 Neurological Department, Zealand University Hospital, Roskilde, Denmark

**Keywords:** Breast cancer, Adjuvant radiotherapy, Sleep disorders, Smartphones, Prospective study

## Abstract

**Background:**

Most breast cancer patients with non-metastatic disease receive adjuvant local or loco-regional radiotherapy. To be scheduled for irradiation may cause distress and fears that can lead to sleep disorders. Few reports focused on sleep problems in patients assigned to radiotherapy. This study evaluates the course of sleep disorders during adjuvant radiotherapy for primary breast cancer and potential risk factors including the use of smartphones or tablets at bedtime.

**Methods:**

The main goal is the evaluation of sleep disorders prior to radiotherapy and after 15 fractions of radiotherapy. A potential effect of habituation to the procedure of radiotherapy can be assumed that will likely lead to improvement (decrease) of sleep disorders. Improvement of sleep disorders (compared to baseline before radiotherapy) is defined as decrease of the severity of sleep disorders by ≥2 points on a patient self-rating scale (0 = no problems; 10 = maximum problems) or decrease of distress caused by sleep disorders by ≥2 points on a self-rating scale (0 = no distress; 10 = maximum distress) or reduction of the dose of sleeping drugs by ≥25%. Additional endpoints include sleep disorders after 5 fractions and at the end of radiotherapy. Moreover, potential risk factors including the use of smartphones or tablets at bedtime are evaluated. Fifty-one patients (48 plus potential drop-outs) are required. With this sample size, a one-sample binomial test with a one-sided significance level of 2.5% has a power of 80% to yield statistical significance, if the rate of patients with improvement of sleep disorders is 25% (rate under the alternative hypothesis) and assuming that a decrease of ≤10% has to be judged as a random, non-causal change in this uncontrolled study setting (null hypothesis).

**Discussion:**

If a decrease of sleep disorders during the course of radiotherapy is shown, this aspect should be included in the pre-radiotherapy consent discussion with the patients. Moreover, identification of additional risk factors will likely lead to earlier psychological support. If the use of smartphones or tablets at bedtime is a risk factor, patients should be advised to change this behavior.

**Trial registration:**

clinicaltrials.gov (NCT04879264; URL: https://clinicaltrials.gov/show/NCT04879264); registered on 7th of May, 2021.

## Background

Breast cancer is one of the most common cancer types in Europe and Northern America [1]. Most of the patients with non-metastatic disease receive adjuvant radiotherapy of the breast or the chest wall [2]. If specific risk factors exist, irradiation may be also indicated for loco-regional lymph nodes. The situation that a breast cancer patient is scheduled for radiotherapy may cause distress due to a sense of menace in view of the technology as well as fear of radiation in general and potential side effects of the planned treatment. A potential consequence of distress and fears can be sleep disorders. Very few studies were published so far that particularly focused on sleep disorders before and during a course of radiation therapy [3.5]. The prevalence of sleep disorders prior to adjuvant radiotherapy for breast cancer ranged between 45 and 48% [3, 4]. Conflicting results were reported regarding sleep disorders during a course of radiotherapy. In a study of breast cancer and prostate cancer patients receiving radiotherapy, sleep disorders occurred mainly before the start of treatment and during the first radiation fractions [5]. Patients appeared to have developed coping strategies during the course of treatment leading to improvement of their sleep disorders. However, in another study of patients with breast or prostate cancer, the patients reported an increase of insomnia during the course of radiotherapy as a result of treatment-related toxicities [6].

This demonstrates that additional studies are required that investigate sleep disorders in breast cancer and prostate cancer patients scheduled for irradiation. If sleep disorders increase during a course of radiotherapy, it is important to identify these patients as soon as possible to be able to offer them early psychological support. Therefore, the knowledge of corresponding risk factors is also very important [4]. If sleep disorders decrease during the course of treatment, radiation oncologists can address this finding and reassure the patients during the informed consent discussion prior to the treatment. In both situations, the patients will benefit from additional studies. The present study evaluates the course of sleep disorders during adjuvant radiotherapy for primary breast cancer and additional potential risk factors.

## Methods and design

This single-arm prospective interventional study conducted at an academic center in Northern Germany investigates sleep disorders prior to and during a course of adjuvant radiotherapy for primary breast cancer. It achieved approval from the ethics committee of the University of Lübeck (reference 21–137) and was registered at clinicaltrials.gov (identifier: NCT04879264).

## Objectives and endpoints

The primary goal of the study is to evaluate sleep disorders in breast cancer patients prior to and during (after 15 fractions/3 weeks) a course of radiotherapy in terms of severity of sleep disorders, distress for the patients and intake of sleeping drugs. These data are used to evaluate the potential effect of habituation to the procedure of radiotherapy and generate hypotheses. Habituation to radiotherapy will likely lead to an improvement (decrease) of sleep disorders during the course of radiotherapy when compared to baseline (prior to radiotherapy). Improvement of sleep disorders is defined as decrease of severity of sleep disorders by at least 2 points on a patient self-rating scale (0 = no problems; 10 = maximum problems) or decrease of distress caused by sleep disorders by at least 2 points on a patient self-rating scale (0 = no distress; 10 = maximum distress) or reduction of the dose of sleeping drugs by ≥25%. The criterion regarding the intake of sleeping drugs was chosen according to another situation of radiotherapy, namely irradiation of painful bone metastases [7]. To obtain the data for these three criteria, patients will complete questionnaires including two self-rating scales and information about intake and doses of sleeping drugs. Additional endpoints include sleep disorders after 5 fractions of radiotherapy, sleep disorders at the end of the radiotherapy course. Moreover, potential risk factors for sleep disorders including the impact of the use of smartphones or tablets at bedtime will be evaluated [3, 4, 8].

### Eligibility criteria

Inclusion criteria include female gender, histologically proven breast cancer, indication for radiotherapy, sleep disorders (at least 2 points on the sleep disorder self-rating scale), Eastern Cooperative Oncology Group (ECOG) performance score of 0–2, age ≥ 18 years, written informed consent, and capacity of the patient to cooperate (including the ability to complete a questionnaire). Exclusion criteria are pregnancy or lactation and limited legal capacity or being under legal supervision.

### Assessments

**Fig. 1:**
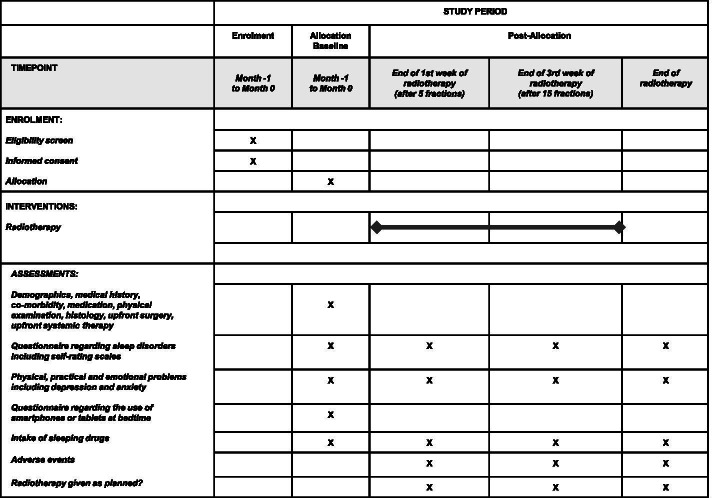
Timeline of enrolment, interventions and assessments of the RADIO-SLEEP study

Parameters assessed prior to radiotherapy include demographics (age, date of birth, gender), medical history, concomitant diseases, physical/practical and emotional problems including depression and anxiety, concomitant medication including sleeping drugs and anticancer treatment, physical examination, histology, upfront surgery, and upfront systemic therapy. The following parameters will be assessed during the course of the study (Fig. 1):

1. Sleep disorders

Sleep disorders will be assessed prior to radiotherapy (baseline), and during (after 5 and 15 fractions) and at the end of the radiotherapy course. Evaluation is based on questionnaires including self-rating scales regarding severity and distress due to sleep disorders and information regarding the intake of sleeping drugs. Moreover, the use of smartphones and tablets at bedtime and other factors such as age, type of breast surgery, physical/practical and emotional problems including depression and anxiety, systemic anticancer treatment, side effects of radiotherapy, body mass index, co-morbidity and other factors causing distress will be correlated to sleep disorders [3, 4, 8].

2. Adverse Events.

Adverse events will be assessed on an ongoing basis according to Common Terminology Criteria for Adverse Events (CTCAE) version 5.0 [9].

### Interventions

Patients receive the same standard radiotherapy for breast cancer following breast-conserving surgery or mastectomy as they would have received without participation in this study. If necessary, any medical care and interventions are permitted during the study.

### Radiotherapy for breast cancer

Most breast cancer patients receive breast-conserving surgery followed by whole-breast irradiation with 40 Gy of hypo-fractionated radiotherapy (5 × 2.667 Gy per week) or 50.4 Gy of conventional fractionation (5 × 1.8 Gy per week) [2]. Patients younger than 51 years and those older than 50 years with increased risk of developing a local recurrence receive a boost of 5 × 2.0 Gy to the tumor bed. After mastectomy, radiotherapy of the chest wall is indicated in the following situations: pT4 or pT3 pN0 R0 and risk factors, incomplete resection without re-resection, involvement of > 3 axillary lymph nodes, involvement of 1–3 axillary lymph nodes plus risk factors [2]. In addition to whole-breast irradiation, radiotherapy of loco-regional lymph nodes is recommended for involvement of > 3 axillary lymph nodes and for involvement of 1–3 axillary lymph nodes plus risk factors. If the treatment volume includes lymph nodes, the preferred dose-fractionation regimen is 50.4 Gy in 28 fractions of 1.8 Gy [2].

### Possible acute side effects of radiotherapy

Radiotherapy can be associated with side effects such as dermatitis, breast edema, esophagitis, pneumonitis, cardia arrhythmia, hypothyroidism, loss of appetite, nausea/vomiting, fatigue and pancytopenia. In case of a grade 3 toxicity according to CTCAE criteria version 5.0 [9], radiotherapy may be delayed for up to 7 days without consequences. If it is delayed for more than 7 days, the coordinating physician of the study must be informed.

### Questionnaires

The patients will be asked to complete a questionnaire prior to radiotherapy, after 5 and 15 fractions of radiotherapy, and at the end of their radiotherapy course (which may be also after 15 fractions). The questionnaire, which is available in full in German only, includes three questions regarding health and general well-being, ten questions regarding distress in everyday life during the last week, and 16 questions regarding symptoms and discomfort during the last week including sleep disorders. In case of sleep disorders, the patients are asked to complete a self-rating scale regarding the severity of their sleep disorders ranging from 0 to 10 (0 = no problems; 10 = maximum problems) and another self-rating scale regarding the distress caused by the sleep disorders also ranging from 0 to 10 (0 = no distress; 10 = maximum distress). In addition, the patients are asked to indicate whether they use sleeping drugs and, if applicable, the type and dose of these drugs. In addition, the patients are asked to answer three general questions regarding their sleep disorders (type of sleep disorders, impact on daily life, and reaction of their social environment). Moreover, the patients who use smartphones or tablets are asked to answer 11 questions regarding the use of these devices in general and at bedtime.

### Sample size calculations

The main goal of the study is to evaluate sleep disorders in breast cancer patients prior to and during a course of radiotherapy in terms of severity of sleep disorders, distress caused by sleep disorders and use of sleeping drugs. This will allow to evaluate the potential effect of habituation to radiotherapy during the course of treatment and generate hypotheses. Forty-eight patients with documented start of radiotherapy, a documented completed questionnaire at baseline, and at least one completed questionnaire during radiotherapy are subjected to statistical analysis. Assuming that 5% of patients do not fulfil the requirements, 51 patients should be enrolled. This sample size is set at the maximum that is deemed achievable in this study within the timeframe given the size of the target population.

With this sample size, a one-sided exact test for one binomial population with a significance level of 2.5% has a power of 80% to yield statistical significance if the rate of patients with improvement (decrease) of sleep disorders during the radiotherapy compared to baseline is 25% (rate under the alternative hypothesis) and specifying a decrease of 10% as non-causal change in this uncontrolled study setting (null hypothesis). With an improvement rate of at least 28% under the alternative hypothesis, the statistical test with this sample size achieves a power of at least 90% (calculated with StatXact, Version 11, Statistical software for exact nonparametric inference, Cytel Software, Cambridge, MA, USA).

### Statistical methods for investigated endpoints

The efficacy analysis will be performed on the modified Full Analysis Set defined as all patients receiving radiotherapy with a completed questionnaire at baseline and at least one completed questionnaire after start of treatment. Similar to a previous study, the statistical analyses are mainly descriptive and exploratory [10]. Sleep disorders at baseline and during radiotherapy will be rated using two patient self-reporting scales (sleep disorders and distress) and intake of sleeping drugs. To evaluate the rate of patients with decrease of sleep disorders after 15 fractions of radiotherapy (generally at the end of the third week of treatment), the dichotomized composite endpoint (decrease of severity of sleep disorders, decrease of distress caused by sleep disorders, reduction of the dose of sleeping drugs) is considered, even if patients discontinue radiotherapy prematurely (treatment policy estimand). The number of missing data will be reported. The point estimate of the rate of decrease and the associated 95% confidence interval will be presented. To test whether the rate of decrease is significantly > 10%, the one-sided binomial test at a one-sided 2.5% significance level will be applied.

To further assess the impact of other factors on the primary endpoint, stratified analyses will be conducted. Factors of particular interest include sleep disorders at baseline and use of smartphones or tablets at bedtime. Furthermore, a logistic regression model including the sleep disorder rating scale at baseline, use of smartphones or tablets at bedtime and other factors such as age, type of breast surgery, systemic anticancer treatment, body mass index and co-morbidity will be fitted to identify significant predictive factors for decrease of sleep disorders. Adjusted odds ratios and 95% confidence interval (Wald χ2) will be derived.

In addition, each component of the primary endpoint will be subjected to statistical analyses to evaluate sleep disorders during the study using descriptive statistical methods. These analyses allow detailed assessments of the scales over time considering potential decrease and increase in sleep disorders. For graphical visualization, Spaghetti-plots and Box-Whisker diagrams will be provided. Changes from baseline will be considered and subjected to descriptive analyses. Friedman and Wilcoxon-signed rank tests may be applied to compare study visits. Cumulative distribution plots of changes from baseline at each visit will be presented; they display a continuous change from baseline on x-axis and the cumulative percentage of patients experiencing that change on y-axis. Analyses will be further stratified by sleeping disorders at baseline and use of smartphones at bedtime. For further exploratory analysis, rates of patients experiencing sleep disorders at each time will be estimated together with associated confidence intervals. These analyses will also be stratified by sleeping disorders at baseline and the use of smartphones at bedtime.

### Data management and monitoring

Similar to a previous study, patients can be identified only by an individual number and their month/year of birth [10]. Data will be handled in accordance with the General Data Protection Regulation and German regulations for radio-protection. Study results will be published in a peer-reviewed journal involving a professional statistician and using the acronym RADIO-SLEEP.

## Discussion

Adjuvant radiotherapy is mandatory after breast-conserving surgery for primary breast cancer and is indicated also after mastectomy if the patients have risk factors for a local or loco-regional recurrence [2]. The situation that a patient has to undergo radiation treatment can cause psychological distress, which is often associated with sleep problems. Occurrence of sleep disorders varies between different types of cancer. In a previous study, patients with breast cancer reported significantly higher rates of sleep disorders, sleep disturbance and fatigue than patients with prostate cancer [11]. Moreover, in a post-hoc analysis of a randomized trial including 823 patients with different types of cancer (49% breast cancer patients) receiving chemotherapy, the proportion of patients with symptoms of insomnia and patients who met the criteria of insomnia syndrome were about three times higher than in the general population [12]. In this study, breast cancer patients reported higher number of sleep disorders than patients with other types of cancer.

Uncertainty exists whether sleep disorders decrease during a course of anticancer treatment due to an effect of habituation to the treatment procedures, or whether disorders increase due to treatment-related side effects and associated clinical symptoms. In a study of breast cancer patients, insomnia was significantly mediated by acute toxicities of chemotherapy or radiotherapy [6]. Since the risk of radiation-related side effects likely decreases with the use of modern high-precision radiotherapy techniques, the effect of habituation to the procedures of radiotherapy during a course of treatment may become stronger resulting in a decrease of sleep problems. This hypothesis is supported by the study of Thomas et al. that investigated changes in sleep during a course of radiotherapy and up to 6 months thereafter in patients with breast cancer or prostate cancer [5]. Both patient groups reported most sleep disorders before and during the first part of their radiation treatment. Additional studies are required to properly answer the question whether sleep problems increase or decrease during a course of radiotherapy. The RADIO-SLEEP study evaluates a potential decrease of sleep disorders due to habituation to radiotherapy in patients irradiated for primary breast cancer. Decrease of sleep disorders will be measured using a composite endpoint considering severity of sleep disorders, distress caused by sleep disorders and the use of sleeping drugs. Data after 15 fractions/3 weeks of radiotherapy will be compared to the data at baseline before radiotherapy (primary endpoint). The data are obtained from questionnaires completed by the patients, which included two self-rating scales and information about intake of sleeping drugs.

Moreover, the RADIO-SLEEP study investigates potential risk factors for sleep disorders. In a retrospective study of 175 breast cancer patients receiving adjuvant radiotherapy, occurrence of sleep disorders was significantly associated with higher distress scores, need for psychological support, and greater numbers of emotional, physical or practical problems [4]. Younger age was previously reported to be significantly associated with insomnia in the above-mentioned post-hoc analysis of patients receiving chemotherapy for different cancer types [11]. In a study of breast cancer patients treated with aromatase inhibitors, younger age showed a trend toward increased insomnia [13]. In another study of breast cancer patients, a high insomnia severity index was more common in patients receiving adjuvant chemotherapy than in those receiving adjuvant radiotherapy [14].

A particular focus of the RADIO-SLEEP study is on the impact of electronic media on sleep disorders. The patients complete a questionnaire regarding the use of such devices in general and specifically at bedtime. Negative associations of the use of electronic media at bedtime on sleep quality have been reported for adolescents and adults in general but not particularly for cancer patients [15–19]. Therefore, the RADIO-SLEEP study includes the use of smartphones or tablets at bedtime as a potential risk factor for sleep disorders in the investigated cohort of breast cancer patients.

If the RADIO-SLEEP study demonstrates a decrease of sleep disorders during the course of adjuvant radiotherapy for primary breast cancer, this aspect should be included in the pre-radiotherapy consent discussion with the corresponding patients. Moreover, if additional risk factors for sleep disorders are identified, this will likely lead to earlier psychological support for the patients. If the use of smartphones or tablets at bedtime is identified as a significant risk factor, patients should be advised to change this behavior.

## Data Availability

Not applicable, since data were not yet generated. The study was registered at clinicaltrials.gov (NCT04879264).

## Abbreviations

CTCAE: Common Terminology Criteria for Adverse Events
ECOG: Eastern Cooperative Oncology Group
RADIO-SLEEP: Sleep disorders prior to and during a course of radiotherapy for breast cancer and the potential impact of smartphones
